# Differentiated service delivery for people using second‐line antiretroviral therapy: clinical outcomes from a retrospective cohort study in KwaZulu‐Natal, South Africa

**DOI:** 10.1002/jia2.25802

**Published:** 2021-10-28

**Authors:** Lara Lewis, Yukteshwar Sookrajh, Kelly Gate, Thokozani Khubone, Munthra Maraj, Siyabonga Mkhize, Lucas E. Hermans, Hope Ngobese, Nigel Garrett, Jienchi Dorward

**Affiliations:** ^1^ Centre for the AIDS Programme of Research in South Africa (CAPRISA) University of KwaZulu–Natal Durban South Africa; ^2^ eThekwini Municipality Health Unit Durban South Africa; ^3^ Bethesda Hospital uMkhanyakude District, KwaZulu‐Natal South Africa; ^4^ Department of Family Medicine University of KwaZulu‐Natal Durban South Africa; ^5^ Department of Medical Microbiology University Medical Center Utrecht (UMCU) Utrecht The Netherlands; ^6^ Wits Reproductive Health and HIV Institute (Wits RHI) University of the Witwatersrand Johannesburg South Africa; ^7^ Discipline of Public Health Medicine, School of Nursing and Public Health University of KwaZulu‐Natal Durban South Africa; ^8^ Nuffield Department of Primary Care Health Sciences University of Oxford Oxford UK

**Keywords:** antiretroviral therapy, differentiated service delivery, HIV, retention in care, second line

## Abstract

**Introduction:**

Evidence is needed to guide the inclusion of broader groups of people living with HIV (PLHIV) in differentiated service delivery (DSD) programmes. We assessed treatment outcomes among PLHIV on second‐line regimens in a community antiretroviral therapy (ART) delivery programme, compared to those who remained at clinics.

**Methods:**

Using data from 61 public clinics, we did a retrospective cohort study among PLHIV receiving second‐line ART following rollout of the Centralized Chronic Medicines Dispensing and Distribution (CCMDD) programme in KwaZulu‐Natal, South Africa. We included PLHIV from the timepoint when they were first eligible, though not necessarily referred, for community ART within CCMDD and followed them for 18 months. We used multivariable logistic regression to compare 12‐month attrition and viraemia between clients referred for community ART and those remaining in clinic care.

**Results:**

Among 209,744 PLHIV aged ≥ 18 years who collected ART between October 2016 and December 2018, 7511 (3.6%) received second‐line ART. Of these, 2575 (34.3%) were eligible for community ART. The median age was 39.0 years (interquartile range 34.0–45.0) and 1670 (64.9%) were women. Five hundred and eighty‐four (22.7%) were referred for community ART within 6 months of meeting eligibility criteria. Overall, 4.5% [95% confidence interval (CI) 3.0–6.6%] in community ART and 4.4% (95% CI 3.5–5.4%) in clinic care experienced attrition at 12 months post eligibility for community ART. Two thousand one hundred and thirty‐eight (83.0%) had a viral load recorded 6–18 months after becoming eligible, and of these, 10.3% (95% CI 7.7–13.3%) in community ART and 11.3% (95% CI 9.8–12.9%) in clinic care had viraemia > 200 copies/ml. In separate regressions adjusted for age, gender, district, time on second‐line ART, nucleoside reverse transcriptase inhibitor backbone and year of eligibility, no differences in the odds of attrition [adjusted odds ratio (aOR) 1.02, 95% CI 0.71–1.47] or viraemia (aOR 0.91, 95% CI 0.64–1.29) were observed between those in community ART and those remaining in clinic care.

**Conclusions:**

We found good outcomes among PLHIV who were stable on second‐line regimens and referred for community ART. Efforts to expand DSD access among this group should be prioritized.

## 1 INTRODUCTION

South Africa has the largest antiretroviral therapy (ART) programme globally with more than 5 million clients receiving ART [1]. In September 2016, the country adopted the policy of universal test and treat, which aims to provide ART to all 7.8 million people living with HIV (PLHIV) regardless of CD4 count [2]. To efficiently achieve universal ART and the UNAIDS 95‐95‐95 targets, the country has implemented the Centralized Chronic Medicines Dispensing and Distribution (CCMDD) programme [3, 4], which has been used to support the rollout of both community‐ and facility‐based differentiated ART delivery [5]. In the community‐based ART delivery programme, PLHIV can collect ART in more convenient locations, such as community pickup points and private pharmacies, rather than at clinics [3, 4, 6, 7]. There is a growing body of evidence supporting the use of such differentiated ART delivery programmes among PLHIV who are stable on first‐line ART [8, 9], in order to provide more efficient, client‐centred care and decongest clinics.

The coronavirus disease 2019 (COVID‐19) pandemic has led to calls to widen access to differentiated ART delivery, to facilitate ART provision through the pandemic, to reduce congestion and thereby COVID‐19 infection risk in clinics and to free up clinic resources to focus on COVID‐19 [10]. One such measure includes expanding eligibility to include people who are stable on second‐line ART. In South Africa, second‐line ART has been included in the CCMDD programme since inception, in contrast to several other countries which restrict differentiated ART delivery to first‐line ART only, and there are little data evaluating differentiated ART delivery outcomes among PLHIV on second‐line ART. These clients may benefit from increased clinic support, because they previously had treatment failure, and second‐line ART regimens are more complex, with worse side effect profiles. Therefore, in this study, we investigate whether, among PLHIV on second‐line ART who were potentially eligible for differentiated care, those who were referred into community ART had similar outcomes to those who continued to collect treatment in public clinics.

## 2 METHODS

### 2.1 Study design and setting

We performed a retrospective cohort analysis using routinely collected anonymized electronic data from between 1 October 2016 and 30 June 2020 in KwaZulu‐Natal, South Africa. We used data from 56 urban clinics run by the eThekwini Municipality Health Unit and data from five rural clinics in the uMkhanyakude District in northern KwaZulu‐Natal. These clinics were selected from existing collaborations and to provide data from both rural and urban settings. KwaZulu‐Natal has an estimated HIV prevalence of 27% among adults aged 15–49 years [11]. ART is provided freely at all public sector clinics using South African National Guidelines, with viral load testing at 6 and 12 months after ART initiation, and annually thereafter [12]. Clients with virological failure, defined as two viral loads >1000 copies/ml more than 2–3 months apart, were recommended to switch to a second‐line ART regimen. Typically, those failing first‐line tenofovir disoproxil fumarate‐based regimens would be switched to zidovudine, lamivudine and lopinavir/ritonavir, while those failing first‐line zidovudine or stavudine‐based regimens would be switched to tenofovir, emtricitabine and lopinavir/ritonavir [12]. In clients with contraindications to tenofovir (e.g. renal impairment) or zidovudine (e.g. anaemia), abacavir was sometimes used.

Prior to April 2020, PLHIV were eligible for CCMDD if they were 18 years or older, had been on the same ART regimen for more than 12 months and if their two most recent viral load measurements were undetectable and taken more than 6 months apart [13]. In addition, clients with tuberculosis (TB), pregnancy, uncontrolled hypertension or diabetes, or other medical conditions requiring regular clinical consultations, were ineligible. Clients referred for community ART would be given 2 months of ART supply at the clinic, with subsequent 2 monthly ART deliveries using the CCMDD programme at a community pickup point of their choice [7]. They would then be reviewed at the clinic every 6 months. Clients who continued to collect ART from the clinic (due to ineligibility for CCMDD, client choice, implementation problems or healthcare workers not deeming community ART delivery to be appropriate) would be seen approximately 2 monthly at the clinic. Although the rollout of CCMDD in KwaZulu‐Natal began in June 2016 [14], we allowed for gradual implementation by starting the study period in October 2016.

### 2.2 Participants

We included PLHIV on second‐line ART meeting CCMDD eligibility criteria captured in the routine clinic database during the period from 1 October 2016 to 31 December 2018. We used the date on which the second suppressed viral load (<200 copies/ml) was taken as baseline, because this was when eligibility could have been first established. We included only those who had at least one clinic visit in the 6 months following eligibility, at which point they could have been referred to either community ART or continued in clinic care. Using the routine clinic data, we excluded individuals who were pregnant or had TB, but it was not possible to identify other medical conditions which may preclude them from inclusion in the community ART programme, such as uncontrolled hypertension or uncontrolled diabetes. Clients were followed up for 18 months after the first point at which eligibility was established.

### 2.3 Data sources and data management

We used de‐identified data extracted from TIER.net, an electronic register in which demographic, clinical and clinic visit data are recorded for all clients initiating and receiving ART in the South African public sector [15]. The register includes data on viral loads, ART regimens, pregnancy and TB status, and referral to the community ART programme. TIER.net data are compared monthly against clinic registers and a subset of clinical charts. Data were checked and cleaned with duplicated records, visits and ART entries removed and ART regimens were rationalized to remove systematic inconsistencies. We did not use the TIER.net lost to follow‐up outcome, as this can be inconsistent [16], and generated our own attrition variable (defined below). Since data were anonymized, data of patients who transferred care to or from another clinic could not be accessed, and ‘silent transfers’ could not be detected. We analysed anonymized data using R 4.0 (R Foundation for Statistical Computing, Vienna, Austria) and SAS, version 9.4 (SAS Institute Inc).

### 2.4 Variables

The primary exposure of interest was a binary variable measuring referral into the community ART programme. PLHIV who were referred within 6 months of eligibility being established were assigned to the community ART group, and those with no referral were assigned to the clinic ART group. Participants in both groups were receiving second‐line ART. Those who were referred to the community ART programme more than 6 months after eligibility was established were assigned to the clinic ART collection group, because of their limited exposure to the community ART programme.

The primary outcomes were attrition and viraemia at 12 months after becoming eligible for community ART. Since the exposure group included clients referred up to 6 months after becoming eligible, the minimum exposure time to community ART at 12 months post eligibility was 6 months. A client was defined as experiencing attrition at 12 months if there was no record of clinic attendance between 12 and 18 months after baseline. Clients who were documented as being transferred to another clinic within 12 months of baseline were assigned a missing value for their attrition outcome as clinic attendance at another clinic could not be matched to baseline data. Patients were defined as having viraemia if they had a viral load > 200 copies/ml 12 months after baseline. We used a window of 6–18 months for the 12‐month viral load because measuring and recording viral loads can be inconsistent in routine healthcare settings [17]. Those with no viral load recorded between 6 and 18 months were assigned a missing value for the viraemia outcome.

Baseline variables that were potentially confounders to the association between community ART referral and outcomes were incorporated in the analysis. These included age, gender, urban or rural district, year in which CCMDD eligibility was established, time on second‐line ART, nucleoside reverse transcriptase inhibitor (NRTI) backbone and most recent CD4 count value, taken within the past 2 years. For those clients who were transferred into the clinic from another facility while already receiving second‐line ART and were missing a second‐line ART start date, we used 30 days before their transfer‐in date as the second‐line ART start date.

### 2.5 Statistical analysis

Baseline and follow‐up characteristics of the cohort were summarized using median and interquartile range (IQR) values for continuous variables and using frequencies and percentages for categorical variables. A Fisher's exact test was used to compare baseline categorical variables of those referred to community ART to those remaining in clinic care. We used generalized estimating equations with a logit‐link and an exchangeable working correlation structure to test the association between the covariates and the outcomes of, first, attrition and second, viraemia, accounting for clinic‐level correlation. Univariable and multivariable regression results are reported. Covariates included in the models were selected based on data availability and clinical significance. As recent CD4 count data were available for only 54.9% of the cohort, it was excluded as a covariate from the main analysis but included in a complete case sensitivity analysis. In a second sensitivity analysis for the attrition outcome, clients who had been transferred to another clinic within 12 months of baseline were included and classified as experiencing attrition. For the viral load outcome, a further sensitivity analysis was performed excluding those who had a follow‐up viral load measured less than 12 months after baseline.

### 2.6 Ethical approval

This work was approved by the University of Kwazulu‐Natal Biomedical Research Ethics Committee (BE646/17), KwaZulu‐Natal Department of Health's Provincial Health Research Ethics Committee (KZ_201807_021), eThekwini Municipality Health Unit and the Bethesda Hospital Ethics Committee, with a waiver for informed consent for analysis of anonymized, routinely collected data.

## 3 RESULTS

### 3.1 Cohort characteristics

**Figure 1:**
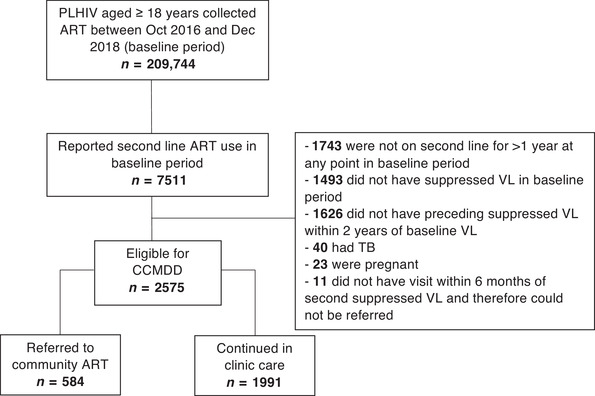
Participant flowchart. ART, antiretroviral therapy; CCMDD, Centralized Chronic Medicines Dispensing and Distribution; PLHIV, people living with HIV; TB, tuberculosis; VL, viral load.

Among 209,744 PLHIV aged ≥ 18 years who collected ART between October 2016 and December 2018, 7511/209,744 (3.6%) received second‐line ART (Figure 1). Of these, 4936/7511 (65.7%) were excluded from analysis as they failed to meet one or more of the community ART programme eligibility criteria captured in the routine clinic database. One thousand six hundred and twenty‐six of these were clients with a suppressed viral load while on second‐line ART, but had no previous suppressed viral load recorded in the previous 6−24 months. A further 11/7511 (0.1%) were excluded as they did not have a clinic visit within 6 months of eligibility at which they could have been referred to the community ART programme. The remaining 2575/7511 (34.3%) were included in the analysis as they were receiving second‐line ART and potentially eligible for the community ART programme during the baseline period of October 2016 and December 2018. The median age of this cohort was 39.0 years (IQR 34.0–45.0) and 1670 (64.9%) were women (Table 1). The majority (*n* = 2389, 92.8%) resided in urban districts.

**Table 1:** Baseline and follow‐up characteristics of clients on second‐line ART who met community ART programme eligibility criteria, split by referral into the community ART programme (*N* = 2575)

		Referred to community ART programme (*n* = 584)	Continued at clinic (*n* = 1991)
*Baseline characteristics*			
Age, median (IQR)		39 (35–45)	39 (34–45)
Age, *n* (%)	<30	55 (9.4)	206 (10.3)
	30–39	246 (42.1)	823 (41.3)
	40–49	203 (34.8)	686 (34.5)
	>50	80 (13.7)	276 (13.9)
Gender, *n* (%)	Female	384 (65.8)	1286 (64.6)
District, *n* (%)	Urban	540 (92.5)	1849 (92.9)
Year of baseline observation, *n* (%)	2016	30 (5.1)	310 (15.6)
	2017	309 (52.9)	977 (49.1)
	2018	245 (42.0)	704 (35.4)
Second‐line protease inhibitor	Lopinavir/ritonavir	581 (99.5)	1980 (99.5)
	Atazanavir	3 (0.5)	11 (0.5)
NRTI backbone^a^	Tenofovir	165 (28.2)	514 (25.8)
	Zidovudine	377 (64.6)	1315 (66.1)
	Abacavir/other^b^	42 (7.2)	162 (8.1)
Months on second‐line ART, median (IQR)		28.5 (18–50)	26 (16–45)
Months since viral load measure preceding baseline viral load, median (IQR)		11 (8–13)	11 (8–13)
Most recent CD4 count at baseline, median (IQR)		449 (260–622)	385 (237–555)
Most recent CD4 count at baseline, *n* (%)	< = 200	34 (11.3)	176 (15.8)
	201–350	70 (23.2)	277 (24.9)
	351–500	55 (18.3)	272 (24.4)
	>500	142 (47.2)	389 (34.9)
	Missing	283	877
Months since most recent CD4 count at baseline, median (IQR)		9 (0–15)	9 (0–15)
Months to community ART referral from baseline	At eligibility	193 (33.1)	
	1–3 months post eligibility	277 (47.4)	
	4–6 months post eligibility	114 (19.5)	
*Follow‐up characteristics*			
Months to viral load follow‐up measurement, median (IQR)		12 (11–12)	12 (11–12)
Missing viral load follow‐up value, *n* (%)		87 (14.9)	350 (17.6)

^a^ Tenofovir typically combined with emtricitabine, zidovudine and abacavir typically combined with lamivudine.

^b^ All but two clients were on abacavir.

ART, antiretroviral therapy; IQR, interquartile range; NRTI, nucleoside reverse transcriptase inhibitor.

Overall, 584/2575 (22.7%) were referred to the community ART programme within 6 months of becoming eligible. The estimated proportion of clients referred into the community ART programme increased with each year in the baseline period from 8.8% in 2016 to 24.0% in 2017 and 25.8% in 2018. The baseline distributions of age, gender, district, NRTI backbone and time on second‐line ART of those referred for community ART were similar to those who remained in clinic care. However, a larger proportion of those receiving community ART had a CD4 count greater than 500 (47.2% vs. 34.9%, *p*<0.001). 166/1991 (8.3%) clients were referred late for community ART at more than 6 months after baseline eligibility and so were included in the clinic care group for analysis.

### 3.2 Attrition

By 12 months, 79/2575 (3.1%) of clients had been transferred to another clinic. Of the remaining 2496, 4.5% [95% confidence interval (CI) 3.0–6.6%] of those receiving community ART for a minimum of 6 months and 4.4% (95% CI 3.5–5.4%) of those in clinic care experienced attrition at 12 months [crude odds ratio (OR) 1.01, 95% CI 0.71–1.45], (Table 2). After adjusting for age, gender, district, time on second‐line ART, NRTI backbone and year of eligibility in a multivariable regression, there was no difference in 12‐month attrition between those referred for community ART and those in clinic care [adjusted odds ratio (aOR) 1.02, 95% CI 0.71–1.47]. In addition, no differences in 12‐month attrition were observed in a sensitivity analysis adjusting for CD4 count and all aforementioned covariates (*n* = 1366, aOR 1.17, 95% CI 0.77–1.77), (Table S1). In a further sensitivity analysis including all clients who were transferred to another clinic, attrition was lower in the community ART group versus clinic care (*n* = 2575, aOR 0.73, 95% CI 0.54–0.99), (Table S2).

**Table 2:** Multivariable logistic regression model of attrition among people living with HIV who are receiving second‐line ART and eligible for referral into the community ART programme (*N* = 2496)

		No recorded visit 12–18 months after baseline, *n* (%) or median (IQR)	OR (95% CI)	Adjusted OR (95% CI)
Age at baseline		39.5 (33–45)	1.00 (0.98–1.02)	1.01 (0.99–1.03)
Gender	Female	75 (4.7)	1.15 (0.83–1.6)	1.21 (0.87–1.67)
	Male	35 (4.0)	1	1
District	Rural	6 (3.4)	0.71 (0.35–1.45)	0.75 (0.35–1.62)
	Urban	104 (4.5)	1	1
Year of baseline observation	2016	14 (4.2)	0.86 (0.56–1.34)	0.87 (0.55–1.39)
	2017	52 (4.1)	0.83 (0.55–1.25)	0.84 (0.55–1.27)
	2018	44 (4.9)	1	1
NRTI backbone at baseline	Tenofovir	28 (4.2)	1.00 (0.63–1.58)	1.05 (0.64–1.72)
	Abacavir/other	14 (7.0)	1.71 (0.94–3.11)	1.7 (0.94–3.1)
	Zidovudine	68 (4.2)	1	1
Months on second line at baseline		25 (14–46)	1.00 (0.99–1.004)	1.00 (0.99–1.005)
Referred into community ART programme	Yes	26 (4.5)	1.01 (0.71–1.45)	1.02 (0.71–1.47)
	No	84 (4.4)	1	1

ART, antiretroviral therapy; CI, confidence interval; IQR, interquartile range; NRTI, nucleoside reverse transcriptase inhibitor; OR, odds ratio.

### 3.3 Viraemia

A total of 2138 (83.0%) had a follow‐up viral load recorded at a median of 12 (IQR 11–12) months after becoming eligible for community ART. 14.9% in the community ART group and 17.6% of those in clinic care were missing a viral load result (Table 1). At follow‐up, 10.3% (95% CI 7.7–13.3%) of PLHIV referred for community ART compared to 11.3% (95% CI 9.8–12.9%) in clinic care had viraemia (OR 0.89, 95% CI 0.64–1.24), (Table 3). After adjusting for age, gender, district, year of eligibility, time on second‐line ART and NRTI backbone, referral for community ART was not found to be significantly associated with the odds of viraemia (aOR 0.91, 95% CI 0.64–1.29). In separate sensitivity analyses, adjustment for CD4 count in the multivariable regression (*n* = 1143, aOR 1.21, 95% CI 0.75–1.94), (Table S3), and exclusion of clients with a viral load taken before 12 months (*n* = 1111, aOR 0.68, 95% CI 0.43–1.05), (Table S4), did not alter findings. Although not the main objective of this analysis, in the multivariable model, there was an association between an abacavir‐based second‐line regimen and viraemia (aOR 1.78, 95% CI 1.21–2.63).

**Table 3:** Multivariable logistic regression model of viraemia (>200 copies/ml) among people living with HIV who are receiving second‐line ART and eligible for referral into the community ART programme (*N* = 2138)

		Viral load > 200 copies/ml 6‐18 months after baseline, *n* (%) or median (IQR)	OR (95% CI)	Adjusted OR (95% CI)
Age at baseline		39 (33–44)	0.99 (0.97–1)	0.99 (0.97–1.01)
Gender	Female	151 (10.8)	0.94 (0.7–1.27)	1.03 (0.74–1.45)
	Male	85 (11.6)	1	1
District	Rural	11 (7.1)	0.63 (0.51–0.78)	0.83 (0.65–1.05)
	Urban	225 (11.3)	1	1
Year of baseline observation	2016	26 (8.9)	0.67 (0.42–1.06)	0.66 (0.38–1.13)
	2017	114 (10.6)	0.83 (0.62–1.1)	0.86 (0.64–1.16)
	2018	96 (12.5)	1	1
NRTI backbone at baseline	Tenofovir	45 (7.9)	0.67 (0.48–0.92)	0.78 (0.55–1.11)
	Abacavir/other	29 (17.3)	1.7 (1.16–2.5)	1.78 (1.21–2.63)
	Zidovudine	162 (11.6)	1	1
Months on second line at baseline		22 (16–36.5)	0.99 (0.99–1)	1.00 (0.99–1.00)
Referred into community ART programme	Yes	51 (10.3)	0.89 (0.64–1.24)	0.91 (0.64–1.29)
	No	185 (11.3)	1	1

ART, antiretroviral therapy; CI, confidence interval; IQR, interquartile range; NRTI, nucleoside reverse transcriptase inhibitor; OR, odds ratio.

## 4 DISCUSSION

In this retrospective cohort study of 61 public sector clinics in South Africa, we found that among PLHIV receiving second‐line ART, those who were referred into a community differentiated ART delivery programme had comparable retention in care and viral load outcomes to those who continued to collect ART in clinics. While these data were collected before the COVID‐19 pandemic, it has implications for countries which are looking to expand access to differentiated ART delivery as part of efforts to continue ART provision during COVID‐19, and beyond.

There are few data regarding outcomes of people receiving second‐line ART in community differentiated ART delivery programmes, and none that compare outcomes with people who continue treatment at clinics. A cohort study in South Africa assessed outcomes among 165 clients with viraemia who recently resuppressed and were referred into facility‐ or community‐based adherence clubs [18]. The study included 105 clients known to be on second‐line ART. Overall retention in care was 94.8% (95% CI 89.8–97.4%) and viral suppression was 83.9% (95% CI 76.8–88.9%) at 12 months. A study in Mozambique of 699 clients who were on second‐ or third‐line regimens and attending community adherence clubs found very high retention in care at 12 months (98.9%, 95% CI 98.2–99.7%) and 12‐month viral suppression of 85.8% (95% CI 83.1–88.2%) [19]. Although these two studies did not include a comparator group that continued to receive standard care in clinics, results from the differentiated ART delivery groups are similar to retention in care and viral suppression outcomes seen in the community ART programme in our study (95.5% and 89.7%, respectively). In our study, only 34% of people receiving second‐line ART were eligible for CCMDD, largely due to not being on second line for >12 months, or not having a known suppressed viral load in the past year. Eligibility criteria for the adherence clubs in the Mozambican and South African cohorts were less strict than in our cohort, with only 6 months on an ART regimen required [19], and only one suppressed viral load needed [18, 19]. Applying these criteria to our cohort would have enabled a further 1626 clients to be eligible for differentiated ART delivery, and these changes have been adopted for all people on ART in new South African guidelines from March 2020, which also allow longer intervals between community ART pickups and less frequent clinic visits [20]. Selective eligibility criteria may explain some of the good outcomes seen among clients on second line in both clinic care and differentiated ART delivery services. However, these good outcomes may also reflect the fact that burdensome clinic visits could have contributed to clients having originally failed first‐line regimens, and easier access through second‐line community ART may enhance retention and viral suppression.

While our study demonstrates good outcomes for people receiving second‐line ART in CCMDD, we cannot be sure that these findings would hold true under the new less strict eligibility criteria, and in particular with longer intervals between ART collection which require a more robust ART supply chain. During COVID‐19, concerns around ART supply chains, including for second‐line regimens, were more pronounced [21]. Our study has some limitations due to the pragmatic use of programmatic data. Firstly, assignment to the exposure groups was non‐random and selection bias may have occurred. Although our analysis adjusted for available demographic and clinical confounders, unmeasured confounders may have meant that clients who were referred for community ART were more stable than those who continued in clinic care, and therefore more likely to have better outcomes. Our definition of eligibility was limited to using data on eligibility criteria stored in the TIER.net database, which excluded criteria on pre‐existing medical conditions of clients. Consequently, there may have been some clients included in the clinic‐care group in the cohort who were not eligible for community ART. If these participants had poorer clinical outcomes than those eligible for community ART, a comparison of the two groups would be biased towards better outcomes among those in the community ART programme. However, we adjusted for NRTI backbone, which is likely a proxy for co‐morbidity [22], and our result was unchanged. An abacavir‐based NRTI backbone was associated with viraemia, which may reflect the negative impact that co‐morbidities can have on treatment outcomes. We used a 6‐month window for a clinic visit to define retention in care at 12 months [23]. As clients in the community ART programme are expected to return to clinic every 6 months, compared to 2 monthly in the clinic group, our attrition window may have biased against community ART clients. Despite this, we found low levels of attrition in the community ART group. Outcomes were measured 12 months after first eligibility for community ART, meaning our results may not reflect longer term outcomes. One hundred and sixty‐six clients who were referred for community ART more than 6 months after eligibility were assigned to the clinic care group, as they would have had less than 6 months in community ART by 12 months of follow up. Under the alternate hypothesis that outcomes for clients in the community ART programme will be better than those in clinic care, inclusion of these clients in the clinic care group may have biased outcomes in the two groups to be more similar.

Our findings are reassuring that clients who are virally suppressed on second‐line ART can be referred safely into community ART programmes and have good clinical outcomes. For ART programmes where this is not already practiced, our findings should encourage policy changes to allow people receiving second‐line ART to benefit from differentiated ART delivery. This is important both in the context of COVID‐19, to reduce health service use and risk of SARS‐CoV‐2 transmission [10], and for ART programmes in general, as they move towards more client‐centred care [24]. Introducing second‐line ART into community ART programmes requires the addition of new ART supply chains, as second‐line regimens can be more complex than single tablet fixed dose combinations that are commonly used in first‐line ART [25]. We note that the proportion of those eligible who were actually referred for community ART rose slowly with time, but remained low. Anecdotally, clinicians at study clinics were sometimes reluctant to refer people on second line due to a perceived need for increased monitoring, and concerns regarding the supply of second‐line drugs both in clinics and at community pickup points [26]. Therefore, supply chains for second‐line regimens must be guaranteed if they are to be successfully included in community ART programmes. Further work is needed to identify why referrals remained low in our clinics, and also to assess longer term outcomes among larger cohorts, and the impact of the more recent changes to CCMDD, particularly in the context of COVID‐19.

## 5 CONCLUSIONS

In this retrospective cohort study of routinely collected data, we demonstrate that among PLHIV on second‐line ART, those who were referred for a community differentiated ART delivery programme had similar clinical outcomes compared to those who remain in clinic care. While our findings are limited by the potential for unmeasured confounding, they support the use of community ART delivery which may provide a more convenient and efficient service for clients receiving second‐line ART. As this may also reduce the burden on clinic resources constrained by the COVID‐19 pandemic, efforts to accelerate the rollout and strengthen community ART delivery among PLHIV on second‐line ART should continue.

## COMPETING INTERESTS

The authors declare no competing interests.

## AUTHORS' CONTRIBUTIONS

JD and NG conceived the analysis. HN, KG, MD, YS and LH oversaw second‐line ART and CCMDD programme implementation. TK, SM, MD, YS, KG and HN oversaw data collection. LL and JD analysed the data. LL and JD drafted the manuscript. All authors critically reviewed and edited the manuscript and consented to final publication.

## FUNDING

This work was supported by a COVID‐19 Adaptations to Differentiated Service Delivery grant from the International AIDS Society and a Fast Track Cities Implementation Science grant from the International Association of Providers of AIDS Care (IAPAC) (2021‐ISG‐Y1‐10004). JD is supported by the Wellcome Trust (grant number 216421/Z/19/Z).

## DATA SHARING

The data used for this analysis cannot be shared publicly because of legal and ethical requirements regarding use of routinely collected clinical data in South Africa. Researchers may request access to the data from the eThekwini Municipality Health Unit and Bethesda Hospital (contact details obtainable upon request to corresponding author).

## Supporting information


**Table S1**. Multivariable logistic regression model of attrition among people living with HIV who are receiving second‐line ART and eligible for referral into the community ART programme, excluding those missing CD4 count data (N = 1,366)
**Table S2**. Multivariable logistic regression model of attrition among people living with HIV who are receiving second‐line ART and eligible for referral into the community ART programme, including those transferred to another clinic as lost to care (N = 2,575)
**Table S3**. Multivariable logistic regression model of viremia (≥200 copies/ml) among people living with HIV who are receiving second‐line ART and eligible for referral into the community ART programme, excluding those missing CD4 count data (N = 1,143)
**Table S4**. Multivariable logistic regression model of viremia (≥200 copies/ml) among people living with HIV who are receiving second‐line ART and eligible for referral into the community ART programme, excluding those with viral load measured less than 12 months after baseline eligibility (N = 1,111)
