# Supplementary material for: Differentiated service delivery for people using second‐line antiretroviral therapy: clinical outcomes from a retrospective cohort study in KwaZulu‐Natal, South Africa
Source: J Int AIDS Soc. 2021 Oct 28;24(Suppl 6):e25802. doi: 10.1002/jia2.25802 (PMC8554220; doi:10.1002/jia2.25802)
Supplement: Supplementary file 1 — Table S1. Multivariable logistic regression model of attrition among people living with HIV who are receiving second‐line ART and eligible for referral into the community ART programme, excluding those missing CD4 count data (N = 1,366) Table S2. Multivariable logistic regression model of attrition among people living with HIV who are receiving second‐line ART and eligible for referral into the community ART programme, including those transferred to another clinic as lost to care (N = 2,575) Table S3. Multivariable logistic regression model of viremia (≥200 copies/ml) among people living with HIV who are receiving second‐line ART and eligible for referral into the community ART programme, excluding those missing CD4 count data (N = 1,143) Table S4. Multivariable logistic regression model of viremia (≥200 copies/ml) among people living with HIV who are receiving second‐line ART and eligible for referral into the community ART programme, excluding those with viral load measured less than 12 months after baseline eligibility (N = 1,111) [file JIA2-24-e25802-s001.docx]

## **Table S1. Multivariable logistic regression model of attrition among people living with HIV who are receiving second-line ART and eligible for referral into the community ART programme, excluding those missing CD4 count data (N=1,366)**

|  |  | **No recorded visit 12-18 months after baseline, n(%) or median (IQR)** |  | **OR (95% CI)** | **Adjusted OR (95% CI)** |
| --- | --- | --- | --- | --- | --- |
| Age at baseline |  | 40.5 (31-45) |  | 1.01(0.98-1.03) | 1.01(0.98-1.03) |
| Gender | Female | 48 (5.6) |  | 1.14(0.78-1.67) | 1.21(0.82-1.81) |
|  | Male | 24 (4.8) |  | 1 | 1 |
| District | Rural | 4 (4.9) |  | 0.92(0.34-2.53) | 0.96(0.32-2.85) |
|  | Urban | 68 (5.3) |  | 1 | 1 |
| Year of baseline observation | 2016 | 8 (3.6) |  | 0.74(0.40-1.36) | 0.72(0.38-1.37) |
|  | 2017 | 40 (6.0) |  | 1.23(0.78-1.94) | 1.19(0.76-1.87) |
|  | 2018 | 24 (5.0) |  | 1 | 1 |
| NRTI backbone at baseline | Tenofovir | 19 (5.4) |  | 1.15(0.66-2.00) | 1.25(0.65-2.4) |
|  | Abacavir/Other | 11 (10.6) |  | 2.31(1.23-4.36) | 2.42(1.29-4.52) |
|  | Zidovudine | 42 (4.6) |  | 1 | 1 |
| Months on 2^nd^ line at baseline |  | 20 (13.5-40.5) |  | 1.00(0.99-1.01) | 1.00(0.99-1.01) |
| CD4 count at baseline | <200 | 9 (4.6) |  | 0.95(0.5-1.81) | 0.96(0.47-1.94) |
|  | 201-350 | 22 (6.6) |  | 1.41(0.86-2.3) | 1.44(0.81-2.56) |
|  | 351-500 | 16 (5.0) |  | 1.05(0.57-1.92) | 1.10(0.6-2.01) |
|  | >500 | 25 (4.9) |  | 1 | 1 |
| Referred into community ART programme | Yes | 18 (6.1) |  | 1.2(0.79-1.82) | 1.17(0.77-1.77) |
|  | No | 54 (5.1) |  | 1 | 1 |

## **Table S2. Multivariable logistic regression model of attrition among people living with HIV who are receiving second-line ART and eligible for referral into the community ART programme, including those transferred to another clinic as lost to care (N=2,575)**

|  |  | **No recorded visit 12-18 months after baseline, n(%) or median (IQR)** |  | **OR (95% CI)** | **Adjusted OR (95% CI)** |
| --- | --- | --- | --- | --- | --- |
| Age at baseline |  | 40 (33-45) |  | 1.01(0.99-1.02) | 1.01(0.98-1.03) |
| Gender | Female | 131 (7.8) |  | 1.24(0.93-1.65) | 1.34(1.01-1.76) |
|  | Male | 57 (6.3) |  | 1 | 1 |
| District | Rural | 13 (7.0) |  | 0.91(0.64-1.29) | 1.05(0.73-1.51) |
|  | Urban | 175 (7.3) |  | 1 | 1 |
| Year of baseline observation | 2016 | 18 (5.3) |  | 0.55(0.39-0.78) | 0.5(0.34-0.75) |
|  | 2017 | 76 (5.9) |  | 0.56(0.43-0.74) | 0.56(0.42-0.75) |
|  | 2018 | 94 (9.9) |  | 1 | 1 |
| NRTI backbone at baseline | Tenofovir | 41 (6.0) |  | 0.77(0.54-1.08) | 0.82(0.57-1.2) |
|  | Abacavir/Other | 19 (9.3) |  | 1.2(0.72-2.02) | 1.22(0.72-2.08) |
|  | Zidovudine | 128 (7.6) |  | 1 | 1 |
| Months on 2^nd^ line at baseline |  | 24 (15-43.5) |  | 0.998(0.992-1.003) | 1.00(0.99-1.01) |
| Referred into community ART programme | Yes | 35 (6.0) |  | 0.76(0.56-1.04) | 0.73(0.54-0.99) |
|  | No | 153 (7.7) |  | 1 | 1 |

## **Table S3. Multivariable logistic regression model of viremia (>200 copies/ml) among people living with HIV who are receiving second-line ART and eligible for referral into the community ART programme, excluding those missing CD4 count data (N=1,143)**

|  | |  | **Viral load >200, n(%) or median (IQR)** |  | **OR (95% CI)** | **Adjusted OR**  **(95% CI)** |
| --- | --- | --- | --- | --- | --- | --- |
| Age at baseline | |  | 38 (32-42) |  | 0.98(0.96-1.00) | 0.98(0.96-1.00) |
| Gender | Female | | 79 (10.8) |  | 0.94(0.61-1.44) | 0.95(0.61-1.49) |
|  | Male | | 49 (11.9) |  | 1 | 1 |
| District | Rural | | 5 (7.8) |  | 0.73(0.48-1.10) | 1.05(0.54-2.04) |
|  | Urban | | 123 (11.4) |  | 1 | 1 |
| Year of baseline observation | 2016 | | 14 (7.3) |  | 0.49(0.24-1.03) | 0.56(0.27-1.17) |
|  | 2017 | | 64 (11.9) |  | 0.92(0.61-1.39) | 1.05(0.69-1.61) |
|  | 2018 | | 50 (12.1) |  | 1 | 1 |
| NRTI backbone at baseline | Tenofovir | | 18 (6.1) |  | 0.48(0.29-0.81) | 0.57(0.33-0.98) |
|  | Abacavir/ Other | | 17 (19.5) |  | 1.90(1.08-3.37) | 1.95(1.10-3.47) |
|  | Zidovudine | | 93 (12.2) |  | 1 | 1 |
| Months on 2^nd^ line at baseline |  | | 21 (15-33.5) |  | 0.99(0.99-1.00) | 1.00(0.99-1.01) |
| CD4 count at baseline* | <200 | | 22 (13.1) |  | 1.76(1.09-2.85) | 1.54(0.89-2.67) |
|  | 201-350 | | 38 (13.6) |  | 1.81(1.09-3.02) | 1.63(0.94-2.83) |
|  | 351-500 | | 35 (12.7) |  | 1.68(0.96-2.93) | 1.67(0.92-3.03) |
|  | >500 | | 33 (7.9) |  | 1 | 1 |
| Referred into community ART programme* | Yes | | 30 (12.3) |  | 1.15(0.74-1.8) | 1.21(0.75-1.94) |
|  | No | | 98 (10.9) |  | 1 | 1 |

* interaction terms between CD4 count and referral into the community ART programme were not significant

## **Table S4. Multivariable logistic regression model of viremia (>200 copies/ml) among people living with HIV who are receiving second-line ART and eligible for referral into the community ART programme, excluding those with viral load measured less than 12 months after baseline eligibility (N=1,111)**

|  | |  | **Viral load >200, n(%) or median (IQR)** |  | **OR (95% CI)** | **Adjusted OR**  **(95% CI)** |
| --- | --- | --- | --- | --- | --- | --- |
| Age at baseline | |  | 38 (32-44) |  | 0.98(0.96-1.00) | 0.98(0.96-1.01) |
| Gender | Female | | 99 (13.5) |  | 1.10(0.74-1.64) | 1.10(0.73-1.68) |
|  | Male | | 53 (14.0) |  | 1 | 1 |
| District | Rural | | 7 (8.1) |  | 0.64(0.5-0.82) | 0.76(0.58-0.98) |
|  | Urban | | 145 (14.2) |  | 1 | 1 |
| Year of baseline observation | 2016 | | 16 (11.4) |  | 0.69(0.35-1.35) | 0.71(0.37-1.38) |
|  | 2017 | | 79 (13.9) |  | 0.96(0.65-1.43) | 1.03(0.69-1.54) |
|  | 2018 | | 57 (14.2) |  | 1 | 1 |
| NRTI backbone at baseline | Tenofovir | | 32 (10.4) |  | 0.73(0.45-1.17) | 0.83(0.5-1.38) |
|  | Abacavir/ Other | | 21 (22.8) |  | 1.93(1.13-3.31) | 1.95(1.15-3.31) |
|  | Zidovudine | | 99 (13.9) |  | 1 | 1 |
| Months on 2^nd^ line at baseline |  | | 22 (16-39) |  | 0.99(0.98-1.00) | 0.994(0.986-1.003) |
| Referred into community ART programme | Yes | | 27 (10.5) |  | 0.66(0.42-1.03) | 0.68(0.43-1.05) |
|  | No | | 125 (14.7) |  | 1 | 1 |
